# Chromosome-scale, haplotype-resolved assembly of human genomes

**DOI:** 10.1038/s41587-020-0711-0

**Published:** 2020-12-07

**Authors:** Shilpa Garg, Arkarachai Fungtammasan, Andrew Carroll, Mike Chou, Anthony Schmitt, Xiang Zhou, Stephen Mac, Paul Peluso, Emily Hatas, Jay Ghurye, Jared Maguire, Medhat Mahmoud, Haoyu Cheng, David Heller, Justin M. Zook, Tobias Moemke, Tobias Marschall, Fritz J. Sedlazeck, John Aach, Chen-Shan Chin, George M. Church, Heng Li

**Affiliations:** 1 Department of Genetics, Harvard Medical School, Boston, MA USA; 2 Department of Data Sciences, Dana-Farber Cancer Institute, Boston, MA USA; 3 Department of Biomedical Informatics, Harvard Medical School, Boston, MA USA; 4 DNAnexus, Mountain View, CA USA; 5 Google, Mountain View, CA USA; 6 Arima Genomics, San Diego, CA USA; 7 Pacific Biosciences, Menlo Park, CA USA; 8 Dovetail Genomics, Scotts Valley, CA USA; 9 Human Genome Sequencing Center, Baylor College of Medicine, Houston, TX USA; 10 Max Planck Institute for Molecular Genetics, Berlin, Germany; 11 Material Measurement Laboratory, National Institute of Standards and Technology, Gaithersburg, MD USA; 12 Saarland University, Saarbrücken, Germany; 13 Max Planck Institute for Informatics, Saarbrücken, Germany

**Keywords:** Computational biology and bioinformatics, Genetics, Molecular biology, Diseases

## Abstract

Haplotype-resolved or phased genome assembly provides a complete picture of genomes and their complex genetic variations. However, current algorithms for phased assembly either do not generate chromosome-scale phasing or require pedigree information, which limits their application. We present a method named diploid assembly (DipAsm) that uses long, accurate reads and long-range conformation data for single individuals to generate a chromosome-scale phased assembly within 1 day. Applied to four public human genomes, PGP1, HG002, NA12878 and HG00733, DipAsm produced haplotype-resolved assemblies with minimum contig length needed to cover 50% of the known genome (NG50) up to 25 Mb and phased ~99.5% of heterozygous sites at 98–99% accuracy, outperforming other approaches in terms of both contiguity and phasing completeness. We demonstrate the importance of chromosome-scale phased assemblies for the discovery of structural variants (SVs), including thousands of new transposon insertions, and of highly polymorphic and medically important regions such as the human leukocyte antigen (HLA) and killer cell immunoglobulin-like receptor (KIR) regions. DipAsm will facilitate high-quality precision medicine and studies of individual haplotype variation and population diversity.

## Abstract (web summary)

Assembly of phased human genomes is achieved by combining long reads and long-range conformational data.

## Main

Humans contain two homologous copies of every chromosome, and deriving the genome sequence of each copy is essential to correctly understand allele-specific DNA methylation and gene expression, and to analyze evolution, forensics and genetic diseases^1^. However, traditional de novo assembly algorithms that reconstruct genome sequences often represent the sample as a haploid genome. For a diploid genome such as the human genome, this collapsed representation results in the loss of half of heterozygous variations in the genome, may introduce assembly errors in regions diverged between haplotypes and may lead to inflated assembly for species with high heterozygosity^2^. Several algorithms have been proposed to generate haplotype-resolved assemblies, also known as phased assemblies. Early efforts such as FALCON-Unzip^3^, Supernova^4^ and our previous work^5^ used relatively short-range sequence data for phasing and can resolve haplotypes only up to several megabases for human samples. These methods are unable to phase through centromeres or long repeats. FALCON-Phase^6^, which extends FALCON-Unzip, uses Hi-C to connect phased sequence blocks and can generate longer haplotypes, but it cannot achieve chromosome-long phasing. Trio binning^7,8^ is the only published method that can do this, plus the assembly and phasing of entire chromosomes. It uses sequence reads from both parents to partition the offspring’s long reads and then assemble each partition separately. However, trio binning is unable to resolve regions heterozygous in all three samples in the trio and will leave such regions unphased. More importantly, parental samples are not always available—for example, for samples caught in the wild or when parents are deceased. For Mendelian diseases, de novo mutations in the offspring will not be captured and phased with the parents if there are no other heterozygotes nearby. This limits the application of trio binning. Therefore, we currently lack methods that can accurately produce phased assembly for a single individual and keep pace with sequence technology innovations.

**Fig. 1:**
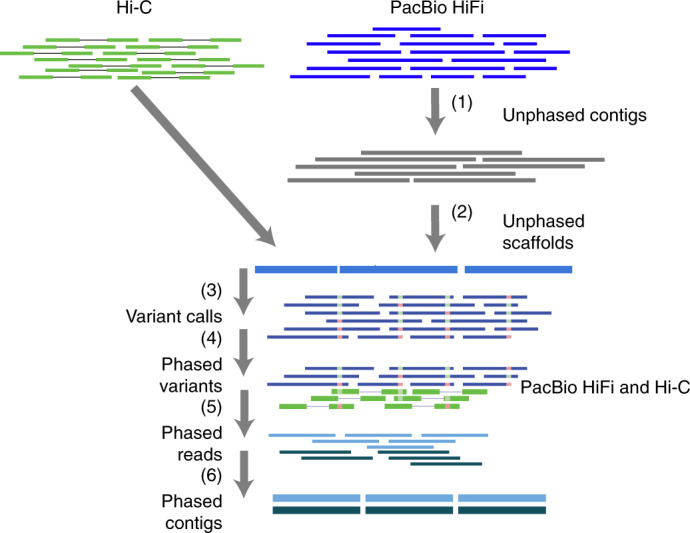
Outline of the phased assembly algorithm, DipAsm. Assemble HiFi reads into unphased contigs using Peregrine (1); group and order contigs into scaffolds with Hi-C data using HiRise/3D-DNA (3D de novo assembly) (2); map HiFi reads to scaffolds and call heterozygous SNPs using DeepVariant (3); phase heterozygous SNP calls with both HiFi and Hi-C data using WhatsHap plus HapCUT2 (4); partition reads based on their phase using WhatsHap (5); assemble partitioned reads into phased contigs using Peregrine (6).

To overcome the limitations in existing methods, we combined recent advances in long-read assembly and Hi-C-based phasing to develop DipAsm, which accurately reconstructs the two haplotypes in a diploid individual using only PacBio’s long high-fidelity (HiFi) reads^9^ and Hi-C data^10^, both at ~30-fold coverage, without any pedigree information (Fig. 1). Starting with an unphased Peregrine^11^ assembly scaffolded by 3D-DNA^12^ or HiRise^13^, our pipeline calls small variants with DeepVariant^14^, phases them with WhatsHap^15^ and HapCUT2 (ref. ^16^), partitions the reads and assembles each partition independently with Peregrine again (Methods). Grouping contigs into chromosome-long scaffolds is necessary for phasing of entire chromosomes by WhatsHap and HapCUT2.

We demonstrate our method on four human genomes: PGP1 from the Personal Genome Project, HG002 and NA12878 from the Genome in a Bottle dataset^17,18^ (GIAB) and HG00733 from the Human Genome Structural Variation Consortium (HGSVC)^19^. We produced HiFi data for the PGP1 genome and Hi-C data for HG002 and HG00733, and assembled the samples with DipAsm (Table 1). For HG002, we also generated a trio-binning-based assembly with Peregrine using parental Illumina reads (Trio Peregrine in Table 1) and obtained a published Trio Canu assembly^9^ for comparison (Table 1). All HG002 assemblies took the same HiFi data as input. For HG00733, we downloaded a FALCON-Phase assembly^6^ and a recent assembly assembled from HiFi and Strand-seq^20^. The Strand-seq assembly and our assembly use the same HiFi data, while the FALCON-Phase assembly uses noisy continuous long read (CLR) and a different Hi-C dataset.

**Table 1:** Assembly statistics

Sample	HG002 (NA24385)			NA12878	PGP1	HG00733		
Assembly algorithm	Trio Canu	Trio Peregrine	DipAsm	DipAsm	DipAsm	DipAsm	Strand-seq	Falcon-Phase
Long-read coverage	29.7 (HiFi)			30.1 (HiFi)	23.9 (HiFi)	33.4 (HiFi)		93.0 (CLR)
Long-read N50 (bp)	13,480			10,004	12,974	11,769		33,090
Hi-C read coverage			38.5	44.8	261.7	35.5		67.1
Scaffolding			3D-DNA	HiRise	HiRise	3D-DNA		
Paternal/maternal contig size (Gbp)	2.96/3.04	2.81/2.88	2.98/2.97	2.97/2.97	2.98/2.98	2.93/2.93	2.90/2.90	2.89/2.89
Paternal/maternal contig NG50 (Mbp)	15.5/18.3	16.6/15.2	25.2/24.3	19.6/18.7	15.1/18.4	25.2/26.2	28.5/23.6	22.3/22.3
Paternal/maternal contig NGA50 (Mbp)	10.2/12.8	11.0/10.6	14.3/13.5	12.7/12.1	10.3/11.0	16.0/16.6	15.8/15.8	14.3/13.7
Phasing switch/Hamming error rate (%)	0.38/0.23	0.38/0.31	0.50/0.49	0.15/2.13	0.21/1.63	0.16/0.60	0.30/0.70	0.43/35.8
SNP/INDEL false-positive rate (×10^−6^)	1.9/31.6	2.6/32.0	2.4/27.7	2.0/4.2	–	–	–	–
SNP/INDEL false-negative rate (%)	4.31/5.85	3.28/5.00	0.36/2.09	0.56/1.22	–	3.32/–	4.00/–	7.89/–
SV sensitivity/precision (%)	90.7/92.8	90.6/92.6	93.4/92.6	–	–	–	–	–

HiFi read N50: 50% of HiFi reads are longer than this number. Contig NG50: minimum contig length needed to cover 50% of the known genome (GRCh38). Contig NGA50: similar to NG50 but based on contig alignment lengths to GRCh38 rather than contig sizes. Phasing switch error rate: percentage of adjacent SNP pairs wrongly phased. Phasing Hamming error rate: percentage of SNPs wrongly phased in comparison to true phases. Gbp, giga base pairs. Mbp, mega base pairs.

From sample HG002, we generated a phased de novo assembly of 5.95 gigabases (Gb) in total, including both parental haplotypes. Half of the assembly is contained in contigs of length ~25 Mb (that is, N50), achieving better contiguity than trio-binning-based assemblies. The scaffold N50 for each parent is >130 Mb. In comparison to GIAB’s single-nucleotide polymorphisms (SNPs) phased by trio, our phasing disagrees at only 0.49% of heterozygous SNPs. This low Hamming error rate over the whole genome suggests we have phased almost every chromosome into maternal and paternal haplotypes, and that the switch errors occurring result in only small local errors in phasing of a small fraction of variants.

To evaluate the consensus accuracy of our assembly, we ran the dipcall pipeline^21^ to align the phased contigs of HG002 against the human reference genome, called SNPs and short insertions and deletions (INDELs) from the alignment and then compared the assembly-based variant calls to GIAB truth calls. Out of the 2.36-Gb confident regions in GIAB, our de novo assembly yields 5,753 false SNP alleles (0.19% of called SNPs) and 65,302 false INDEL alleles (11.86% of called INDELs); 77% of INDEL errors are 1-base-pair (bp) deletions, consistent with a previous observation that 1-bp deletion is the major error mode for this dataset^9^. On the assumption that false-positive calls are all consensus errors and not structural assembly errors or contig alignment errors, this gives a per-base error rate of 1.5 × 10^−5^ (which equals (5,753 + 65,392)/(2 × 2.36 × 10^9^)), or Q48 in the Phred scale. Notably, our de novo assembly achieves a consensus accuracy comparable to that of the Arrow-polished Trio Canu assembly. This suggests that signal-based Arrow polishing may not be necessary for HiFi data.

Comparison to GIAB truth data also reveals the phasing power. During assembly, failure to partition reads in heterozygous regions leads to the loss of heterozygotes and thus the elevated false-negative rate in Table 1. On this metric, our Hi-C-based assemblies miss only 0.4% of heterozygous SNPs, around eight times better than trio-binning-based assemblies. Trio binning is less powerful potentially because it is unable to phase a heterozygote when all individuals in a trio are heterozygous at the same site. In addition, trio binning breaks short reads into *k*-mers, which also reduces power in comparison to mapping of full-length, paired-end Hi-C reads in our pipeline.

The dipcall pipeline outputs phased long INDELs along with small variants. Evaluated against the GIAB SV truth set^22^ (v.0.6) with Truvari v.1.3.2, our de novo assembly-based callset shows a sensitivity of 93.4% and precision of 92.6% (Table 1). The sensitivity of trio-binning-based callsets is ~3% lower, consistent with their lower sensitivity on small variants. Nearly all of the putative false-positive calls are low-complexity sequences. We manually inspected some of these false-positive calls from the de novo assembly. In many cases, our long INDEL calls are apparent in both HiFi read alignment and contig alignment but they are often split into multiple INDEL calls that sum to the same length as the GIAB call. Current SV benchmarking tools are unable to match SVs between VCF files when SVs are represented as multiple events in the variant call format (VCF)^22^. Therefore, our precision is probably substantially higher than 92.6% within GIAB SV benchmark regions.

**Fig. 2:**
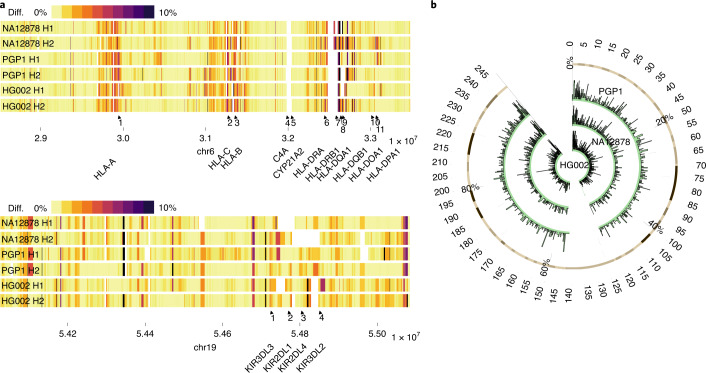
Applications of phased assemblies. **a**, Local sequence divergence in comparison to the reference HLA haplotypes (top) and to the KIR haplotypes (bottom) regions in GRCh38. **b**, SV density (per 100 kb) on chromosome 1 for HG002 (inner), NA12878 (middle) and PGP1 (outer).

We additionally ran RepeatMasker^23^ on SV insertion sequences (9.1 Mb total length) and discovered that 831, 540 and 2,303 of these are within LINEs (long interspersed nuclear element), LTRs (long terminal repeats) and SINEs (short interspersed nuclear elements), respectively. There are 123 microsatellites, 3,582 simple repeats and 270 low-complexity sequences. We also found 21 inversions relative to the reference genome in these HG002 haplotigs (maximum length 25 kb, average length 5 kb). A subset of SVs called from our haplotype assemblies are analyzed in Fig. 2b.

Our HG00733 assembly has similar contiguity to the Strand-seq assembly. Evaluated against the phased SNP calls generated by the HGSVC project^19^, our assembly has slightly lower phasing error rate and phases more heterozygous SNPs. It is worth noting that the HGSVC calls are not curated. Some of the false negatives in the table may be false positives by HGSVC. We also cannot estimate false-positive rates because HGSVC does not provide confident regions. Both the Strand-seq assembly and our assembly can phase entire chromosomes but the FALCON-Phase assembly cannot, as indicated by the 35.8% Hamming error rate. The FALCON-Phase assembly swaps large blocks of haplotypes between the two phases.

We assembled two further human genomes, NA12878 and PGP1, with DipAsm. We could achieve chromosome-long phasing, albeit with a shorter read length of NA12878 and lower read coverage of PGP1. Compared again to GIAB, the NA12878 assembly has even better consensus accuracy, measured at Q55 in GIAB confident regions. Notably, the raw HiFi base quality of NA12878 and HG002 is similar. To understand why NA12878 has better consensus, we counted distinct 31-mers in both assemblies and HiFi reads. We found for NA12878 that 3.63% of 31-mers occurring at least three times in reads are absent from the assembly but, for HG002, the percentage rises to 6.35%. Given that the completeness of NA12878 and HG002 is similar, the higher percentage suggests that there are more recurrent sequencing errors in HG002, which could explain the lower consensus accuracy of HG002.

The HLA and KIR regions are among the most polymorphic in the human genome. Our phased assemblies can reconstruct most of these regions with two contigs for each haplotype. Based on the pattern of local sequence divergence (Fig. 2a), we can see that the two haplotypes in each individual are distinct from one another. Such regions can be faithfully assembled only when we phase through the entire regions.

We present a method to generate a phased assembly for a single human individual or, potentially, a diploid sample of other species. It accurately produces chromosome-long phasing using only two types of input data: HiFi and Hi-C. In comparison to other published single-sample phased assembly algorithms, our method is capable of chromosome-long phasing. While Strand-seq, in combination with HiFi, has recently been used to phase entire chromosomes as well^20^, Hi-C is easier to produce and more widely used. In comparison to trio binning, our method is not restricted to samples having pedigree data and can phase de novo mutations. It gives more contiguous assembly and phases a larger fraction of the genome for human samples. Meanwhile, our assembly strategy is not without limitations. First, relying on accurate SNP calls from long reads and using Peregrine for assembly, our pipeline does not work with noisy long reads at present. It is possible to switch to a noisy read assembler and to add Illumina data for SNP calling, but assembly accuracy may be reduced due to the elevated sequencing error rate. Second, starting with an unphased assembly, we may miss highly heterozygous regions involving long SVs, as demonstrated in our previous works on small genomes^5,8^. A potential solution is to retain heterozygous events in the initial assembly graph and to scaffold and dissect these events later to generate a phased assembly. Nevertheless, our improved de novo method sets a milestone. Its ability to generate phased assemblies without using a reference sequence will enable the unbiased characterization of human genome diversity and construction of a comprehensive human pangenome, which are currently goals of the Human Genome Reference Project. The ability to accurately resolve highly polymorphic regions of biological importance, such as HLA and KIR, will further the goals of precision medicine.

## Methods

### PacBio circular consensus sequencing for PGP1

Library preparation: genomic DNA was converted into a SMRTbell library as previously described^9^, but with several modifications to generate slightly larger inserts. Specifically, gDNA was sheared using MegaruptorR from Diagenode with the 30-kb shearing protocol using a long hydropore cartridge. Before library preparation, the size distribution of sheared DNA was characterized on the Agilent Femto Pulse System. A sequencing library was constructed from this sheared gDNA using the SMRTbell Template Prep Kit v.1.0 (Pacific Biosciences, no. 100-259-100). To tighten the size distribution of the SMRTbell library, it was size fractionated using the SageELF System from Sage Science. Approximately 4 µg of the SMRTbell library was prepared with loading solution/Marker40; next, the sample was loaded onto a 0.75% agarose 10–40-kb gel cassette and size fractionated using a run target size of 7,000 bp set for elution well 12. A total of 8 µg was fractionated on two cassettes. Fractions having the desired size distribution range were identified on the Agilent Femto Pulse System. Fractions centered at 11 kb were pooled to generate an 11-kb library, and those centered at 16 kb were pooled to create a 16-kb library. Both libraries were used for sequencing.

Sequencing: sequencing reactions were performed on the PacBio Sequel System with Sequel Sequencing Kit 3.0 chemistry. The samples were pre-extended without exposure to illumination for 12 h to enable transition of the polymerase enzymes into the highly processive strand-displacing state, and sequencing data were collected for 24 h to ensure maximal yield of high-quality HiFi reads. In addition, sequencing reactions were also performed on the PacBio Sequel II System using Sequel II Sequencing Kit 1.0 chemistry. On the Sequel II system, data collection was extended to 30 h to ensure suitable amounts of data.

### Hi-C sequencing for HG002 and HG00733

A Hi-C library was generated on HG002 and HG00733 by Arima Genomics using a modified version of the Arima-HiC kit. Briefly, the current Arima-HiC kit (no. A510008) utilizes two restriction enzymes for simultaneous chromatin digestion. In the modified protocol, four restriction enzymes were deployed to enable more uniform per-base coverage of the genome while maintaining the highest long-range contiguity signal, thereby benefiting analyses such as variant discovery, base polishing, scaffolding and phasing. After modified chromatin digestion, digested ends were labeled, proximally ligated and then proximally ligated DNA was purified. Following the modified Arima-HiC protocol, Illumina-compatible sequencing libraries were prepared by first shearing purified Arima-HiC ligation products and then size selecting DNA fragments using SPRI beads. The size-selected fragments containing ligation junctions were enriched using enrichment beads provided with the Arima-HiC kit, and converted into Illumina-compatible sequencing libraries using the Swift Accel-NGS 2S Plus kit (no. 21024) reagents. After adapter ligation, DNA was PCR amplified and purified using SPRI beads. Purified DNA underwent standard quality control (quantitative PCR and Bioanalyzer) and was sequenced on HiSeq X following the manufacturer’s protocols.

### Phased sequence assembly

We ran Peregrine v.0.1.5.2 with the following command line: ‘peregrine asm reads.lst 24 24 24 24 24 24 24 24 24 --with-consensus --shimmer-r 3 --best_n_ovlp 8--output asm’, where file ‘reads.lst’ gives the list of input read files and directory ‘asm’ holds the output assembly. We mapped Hi-C reads to contigs with BWA-MEM v.0.7.17 and scaffolded the Peregrine contigs with juicer v.1.5 and 3D-DNA v.180922. We preprocessed data with ‘juicer.sh -d juicer -p chrom.sizes -y cut-sites.txt -z contigs.fa -D’, where file ‘cut-sites.txt’ was generated using the generate_site_positions_Arima.py script, which outputs merged_nodups.txt. The scaffolds were produced with ‘run-asm-pipeline.sh -m haploid contigs.fa merged_nodups.txt’. We then called small variants using DeepVariant v.0.8.0 with the pretrained PacBio model. We mapped Hi-C reads to the scaffolds and ran HapCUT2 v.1.1 over heterozygous SNP sites to obtain sparse phasing at the chromosome scale. The resulting haplotypes were then combined with PacBio HiFi data using WhatsHap v.0.18, with default parameters, to generate fine-scale, chromosome-long phasing. We partitioned HiFi reads based on the phases of SNPs residing on these reads, and ran Peregrine again for reads on the same haplotype from the same scaffold. This provided the final phased assembly.

### Evaluation of variant calling accuracy

For GIAB samples HG002 and NA12878, we compared small variant calls to GIAB v.3.3.2 with RTG’s vcfeval v.3.8.4. We extracted allelic errors with the ‘hapdip.js rtgeval’ script from the syndip pipeline^21^. For sample HG002, we used Truvari v.1.3.2 to evaluate long INDEL accuracy against GIAB SV v.0.6. We specified the option ‘--passonly --multimatch’ to skip filtered calls in the GIAB VCF and to allow matching of base calls to multiple comparison calls, and vice versa. Increasing evaluation distance from the default 500 to 1,000 with ‘-r 1,000’ only marginally improved precision, from 92.6 to 93.3%.

### Reporting Summary

Further information on research design is available in the Nature Research Reporting Summary in this article.

## Online content

Any methods, additional references, Nature Research reporting summaries, source data, extended data, supplementary information, acknowledgements, peer review information; details of author contributions and competing interests; and statements of data and code availability are available at 10.1038/s41587-020-0711-0.

## Supplementary information

Reporting Summary

## Data Availability

HG002 HiFi reads and the 250-bp parental short reads were acquired from the GIAB ftp site: ftp://ftp-trace.ncbi.nlm.nih.gov/giab/ftp/. HG002 Hi-C (no. accession code no. SRR11016318), HG00733 Hi-C (accesssion code no. SRR11347815) and PGP1 HiFi reads (accession code no. SRR11016319) sequenced by us were deposited with Sequence Read Archive (SRA). NA12878 HiFi (accession code no. SRX5780566), Hi-C (accession no. SRR6675327) and PGP1 Hi-C reads^24^ (accession code no. SRP173234) were downloaded from SRA. The HG00733 Falcon-Phase assembly was obtained from NCBI (accession code no. GCA_003634875.1). Other assemblies and assembly-based variant calls used in this work are publicly available at ftp://ftp.dfci.harvard.edu/pub/hli/whdenovo/. HG00733 phased SNP calls were downloaded from ftp://ftp.1000genomes.ebi.ac.uk/vol1/ftp/data_collections/hgsv_sv_discovery/working/20160704_whatshap_strandseq_10X_phased_SNPs/PUR/.

## Code availability

The complete pipeline is available at https://github.com/shilpagarg/DipAsm.
