# Supplementary material for: Chromosome-scale, haplotype-resolved assembly of human genomes
Source: Nat Biotechnol. 2020 Dec 7;39(3):309–12. doi: 10.1038/s41587-020-0711-0 (PMC7954703; doi:10.1038/s41587-020-0711-0)
Supplement: Supplementary file 1 — Reporting Summary [file 41587_2020_711_MOESM1_ESM.pdf]

Corresponding author(s): Shilpa Garg, Chen-Shan Chin, George M. Church, Heng Li

Last updated by author(s): Jun 12, 2020

Reporting Summary

Nature Research wishes to improve the reproducibility of the work that we publish. This form provides structure for consistency and transparency in reporting. For further information on Nature Research policies, see Authors & Referees and the Editorial Policy Checklist.

Statistics

For all statistical analyses, confirm that the following items are present in the figure legend, table legend, main text, or Methods section.

n/a	Confirmed	
☒	☐	The exact sample size (n) for each experimental group/condition, given as a discrete number and unit of measurement
☒	☐	A statement on whether measurements were taken from distinct samples or whether the same sample was measured repeatedly
☒	☐	The statistical test(s) used AND whether they are one- or two-sided<br>*Only common tests should be described solely by name; describe more complex techniques in the Methods section.*
☒	☐	A description of all covariates tested
☒	☐	A description of any assumptions or corrections, such as tests of normality and adjustment for multiple comparisons
☒	☐	A full description of the statistical parameters including central tendency (e.g. means) or other basic estimates (e.g. regression coefficient) AND variation (e.g. standard deviation) or associated estimates of uncertainty (e.g. confidence intervals)
☒	☐	For null hypothesis testing, the test statistic (e.g. F, t, r) with confidence intervals, effect sizes, degrees of freedom and P value noted<br>*Give P values as exact values whenever suitable.*
☒	☐	For Bayesian analysis, information on the choice of priors and Markov chain Monte Carlo settings
☒	☐	For hierarchical and complex designs, identification of the appropriate level for tests and full reporting of outcomes
☒	☐	Estimates of effect sizes (e.g. Cohen's d, Pearson's r), indicating how they were calculated

Our web collection on statistics for biologists contains articles on many of the points above.

Software and code

Policy information about availability of computer code

Data collection	No software is used for data collection.
Data analysis	Open source software: Peregrine v0.1.5.2, BWA-MEM v0.7.17, minimap2 v2.17, juicer v1.5, 3D-DNA v180922, DeepVariant v0.8.0, HapCUT2 v1.1, WhatsHap v0.18, RaGOO v1.1 and RTG tools v3.8.4.<br>Command lines: https://github.com/shilpagarg/DipAsm

For manuscripts utilizing custom algorithms or software that are central to the research but not yet described in published literature, software must be made available to editors/reviewers. We strongly encourage code deposition in a community repository (e.g. GitHub). See the Nature Research guidelines for submitting code & software for further information.

Data

Policy information about availability of data

All manuscripts must include a data availability statement. This statement should provide the following information, where applicable:

- Accession codes, unique identifiers, or web links for publicly available datasets
- A list of figures that have associated raw data
- A description of any restrictions on data availability

HG002 HiFi reads and the 250bp parental short reads were acquired from the GIAB ftp site ftp://ftp-trace.ncbi.nlm.nih.gov/giab/ftp/. HG002 Hi-C reads (AC:SRR11016318), HG00733 Hi-C (AC:SRR11347815) and PGP1 HiFi reads (AC:SRR11016319) sequenced by us were deposited to SRA. NA12878 HiFi (AC:SRX5780566) and Hi-C reads (AC:SRR6675327), and PGP1 Hi-C reads (AC:SRP173234) were downloaded from SRA. The HG00733 Falcon-Phase assembly was obtained from NCBI (AC:GCA_003634875.1). Other assemblies and assembly-based variant calls used in this work are publicly available at ftp://ftp.dfci.harvard.edu/pub/hli/whdenovo/. HG00733 phased SNP calls were downloaded from ftp.1000genomes.ebi.ac.uk/vol1/ftp/data_collections/hgsv_sv_discovery/working/20160704_whatshap_strandseq_10X_phased_SNPs/PUR/.

Field-specific reporting

Please select the one below that is the best fit for your research. If you are not sure, read the appropriate sections before making your selection.

☒ Life sciences ☐ Behavioural & social sciences ☐ Ecological, evolutionary & environmental sciences

For a reference copy of the document with all sections, see nature.com/documents/nr-reporting-summary-flat.pdf

Life sciences study design

All studies must disclose on these points even when the disclosure is negative.

Sample size	Not applicable as the study does not include any statistical analysis.
Data exclusions	No data was excluded from analysis.
Replication	The method has been evaluated on three human genomes and achieves similar performance and accuracy.
Randomization	Not applicable as no statistical analysis is involved.
Blinding	Not applicable as no data acquisition or statistical analysis is involved.

Reporting for specific materials, systems and methods

We require information from authors about some types of materials, experimental systems and methods used in many studies. Here, indicate whether each material, system or method listed is relevant to your study. If you are not sure if a list item applies to your research, read the appropriate section before selecting a response.

Materials & experimental systems

n/a	Involved in the study	
☒	☐	Antibodies
☒	☐	Eukaryotic cell lines
☒	☐	Palaeontology
☒	☐	Animals and other organisms
☒	☐	Human research participants
☒	☐	Clinical data

Methods

n/a	Involved in the study	
☒	☐	ChIP-seq
☒	☐	Flow cytometry
☒	☐	MRI-based neuroimaging
